# Saliva-Induced Clotting Captures Streptococci: Novel Roles for Coagulation and Fibrinolysis in Host Defense and Immune Evasion

**DOI:** 10.1128/IAI.00307-16

**Published:** 2016-09-19

**Authors:** Kristofer Wollein Waldetoft, Tirthankar Mohanty, Christofer Karlsson, Matthias Mörgelin, Inga-Maria Frick, Johan Malmström, Lars Björck

**Affiliations:** Division of Infection Medicine, Department of Clinical Sciences, Lund University, Lund, Sweden; Albert Einstein College of Medicine

## Abstract

Streptococcal pharyngitis is among the most common bacterial infections, but the molecular mechanisms involved remain poorly understood. Here we investigate the interactions among three major players in streptococcal pharyngitis: streptococci, plasma, and saliva. We find that saliva activates the plasma coagulation system through both the extrinsic and the intrinsic pathways, entrapping the bacteria in fibrin clots. The bacteria escape the clots by activating host plasminogen. Our results identify a potential function for the intrinsic pathway of coagulation in host defense and a corresponding role for fibrinolysis in streptococcal immune evasion.

## INTRODUCTION

Streptococci of groups A, C, and G impose a tremendous disease burden globally, causing severe invasive infections as well as immune sequelae (1, 2), and their molecular interactions with the human host have therefore been extensively studied, mainly in group A streptococci (GAS) (3, 4). Dissecting the pathogenesis of invasive infections, these studies have revealed a number of virulence factors that interact with host systems, including the coagulation and fibrinolytic systems (5–9). For example, these bacteria produce streptokinase, a secreted enzyme that activates human plasminogen into fibrinolytic plasmin (8, 10, 11), a phenomenon that will play a central role in this study.

For the bacteria, however, invasive infections are probably dead ends, and they constitute but a minute proportion of streptococcal infections. For example, in group A streptococci, pharyngitis is almost one thousand times more common, and impetigo and asymptomatic carriage are highly prevalent as well (1, 12). Thus, circulating strains should have a history that includes many episodes of superficial infection and/or carriage and few, if any, episodes of invasive infection. This has consequences for our understanding of streptococcal adaptations, since an organism's adaptations are the result of selection in the history of its lineage. In line with this, it has been argued that some virulence factors may not be adaptations for the severe infections in which they have been studied, but are rather by-products of selection in other contexts (13, 14). In order to understand streptococcal adaptations, it is thus important to investigate how the bacteria interact with host systems in the noninvasive settings where they spent their history (14–17).

Streptococcal pharyngitis is a very common infection, with a yearly incidence in excess of half a billion cases (1, 18). It involves inflammation of the pharynx and tonsils, resulting in an inflammatory exudate (19). Once at the pharyngeal epithelium, the plasma exudate will mix with saliva and the bacteria.

In the present study, we investigate the interactions among these three players—plasma, saliva, and streptococci. We find that saliva activates the plasma clotting system, including the intrinsic pathway of coagulation. The bacteria are entrapped in the clots, but escape by inducing fibrinolysis.

## MATERIALS AND METHODS

### Bacteria and growth conditions.

G45, the strain used in most experiments in this paper, is a group G streptococcus (GGS) strain of Streptococcus dysgalactiae subsp. equisimilis isolated from the pharynx of a patient with pharyngitis at the Royal Brisbane Hospital (Brisbane, Australia). GGS 1 in Fig. 5a is the G45 strain, GGS 2 is strain G67, and GCS is the group C streptococcus strain C17. The group A streptococcus (GAS) strains in Fig. 5a are denoted according to serotype: M1 a is strain AP1, M1 b is strain LA2, M3 is an M3 strain, M6 is AP6, and M49 is NZ131. The Streptococcus parasanguinis strain is FK1, and the Streptococcus oralis strain is FK4 isolated from a healthy carrier. Bacteria were cultured in THY broth (Todd-Hewitt broth [Difco] supplemented with 0.5% [wt/vol] yeast extract [Oxoid]) and harvested in the mid-exponential phase (0.4 < optical density at 620 nm [OD_620_] < 0.5; path length, 13 mm [Thermo Spectronic Genesys 20]) to limit variation across experiments (20).

### Saliva, plasma, NaCl solutions, and calcium measurement.

Saliva was collected from healthy donors in the morning. Donors brushed their teeth, rinsed their mouths thoroughly, waited for at least 30 min, and spat into plastic tubes on ice. The saliva was centrifuged at 16,100 relative centrifugal force (RCF) at 4°C, and the supernatant was sterile filtered (Millex; Millipore). For preparation of the fraction of saliva smaller than 3 kDa (S<3), saliva sterile filtrate was passed through a centrifugal filter with a 3-kDa cutoff (Ultracel −3K; Millipore). For preparation of the fraction larger than 3 kDa (S>3), the retentate from the centrifugal filter was washed and resuspended to the original volume in 145 mM NaCl with 1.7 mM CaCl_2_. Alternatively, the retentate was washed and resuspended to the original volume in 38 mM NaCl with 1.7 mM CaCl_2_. All material was kept on ice until use.

For preparation of normal plasma without anticoagulant, blood was drawn from healthy donors with plastic syringes, and immediately transferred to precooled plastic tubes on ice. The blood was centrifuged at 3,000 RCF at 4°C, and the supernatant was kept on ice until use. The fraction of plasma smaller than 3 kDa (P<3) was prepared with 3-kDa centrifugal filters, as described for S<3. Citrated normal plasma was prepared by centrifugation at 700 RCF at room temperature of blood collected with BD Vacutainer, and the plasminogen-depleted plasma was from Affinity Biologicals. All plasma samples were used at a final concentration of 10% to mimic an inflammatory exudate in the upper airways. This concentration was estimated from glucose concentrations in nasal secretions from individuals with rhinitis and healthy controls (21).

NaCl solutions with ionic strengths of 38 and 145 mM were used to approximate the ionic strengths (of the <3-kDa fractions) of saliva and plasma, respectively. Based on the electrolyte composition of saliva reported in Table 1 in reference 22, using the reported [H^+^] to calculate the equilibrium ratio of HPO_4_^2−^ to H_2_PO_4_^−^, and assuming that the resulting anion gap represents monovalent ions, the ionic strength is approximately 36 mM. Based on the electrolyte composition of plasma reported in reference 23, the ionic strength is approximately 140 mM. In addition, 38 and 145 mM are close to the ionic strengths of synthetic salivas reported in reference 24 and crystalloids used for fluid therapy (23), respectively. Figure 3a shows the activation of factor XII (FXII) for a range of ionic strengths for comparison.

The calcium concentrations in S<3 and P<3 from the donors used in the clotting experiments (with and without bacteria) were determined with a calcium measurement kit (Abcam ab 102505) and found to be 1.7 mM in both types of material from both donors.

Blood for the preparation of normal plasma was drawn from healthy volunteers in accordance with an ethical approval from the regional ethical committee (Regionala Etikprövningsnämnden Lund; Dnr 657/2008).

### Bacterial entrapment and escape experiments.

For visualization, G45 bacteria were washed and diluted to 2 × 10^9^ CFU/ml in TG buffer (10 mM Tris-HCl, pH 7.5, with 5 mM glucose). A 750-μl bacterial suspension was centrifuged at 13,000 RCF for 1 min, the pellet was resuspended in 2.25 ml of saliva or 12.9 mM trisodium citrate dihydrate (henceforth sodium citrate) solution, and 250 μl plasma without anticoagulant was added, together with chloramphenicol (to a final concentration of 10 μg/ml). The samples were incubated at 37°C for 1 h under rotation, gently shaken, and photographed.

For quantification of entrapment using optical density, G45, G67, C17, AP1, LA2, M3, AP6, and NZ131 bacteria were washed and diluted to 2 × 10^9^ CFU/ml in TG buffer. A 300-μl bacterial suspension was centrifuged at 13,000 RCF for 1 min, the pellet was resuspended in 900 μl of saliva or 12.9 mM sodium citrate solution, and 100 μl of plasma without anticoagulant was added. Chloramphenicol was added to all samples (to a final concentration of 10 μg/ml). The samples were vortexed and incubated at 37°C for 1 h on a rotator. Any clots formed were left in the tube, the fluid was transferred to cuvettes, and the optical density at 620 nm was measured (path length, 10 mm [Thermo Spectronic Genesys 20]).

Escape from clots was quantified using the same setup as the entrapment experiments above, with the addition of a chloramphenicol-free treatment. The strains G45, C17, LA2, FK1, and FK4 were used.

For quantification using colony counting, G45 bacteria were washed and diluted to 2 × 10^9^ CFU/ml in TG buffer. A 300-μl bacterial suspension was centrifuged at 13,000 RCF for 1 min, the pellet was resuspended in 810 μl of saliva, and 90 μl of normal citrated plasma, plasminogen-depleted plasma, or plasminogen-depleted plasma with the FXII/plasma kallikrein (PK) inhibitor H–d-Pro–Phe–Arg–chloromethylketone (CMK) (50-μg/ml final concentration [Bachem AG]) was added. Three hundred microliters of each sample was removed (denoted “Start”) and put on ice, and the remaining material was incubated at 37°C for 1 h under rotation. After incubation, 300 μl was withdrawn from the middle of each tube (denoted “Sup.” [clots adhere to the sides of the tubes used in this assay]), and transferred to new tubes, leaving 300 μl of material in the original tubes (denoted “Clot,” whether or not any clots were observed). To each tube (Start, Sup., and Clot), 300 μl 4-mg/ml trypsin (Sigma) in 0.05 M KH_2_PO_4_ with 0.005 M EDTA (pH 6.1) was added, and the samples were incubated at 37°C for 20 min under rotation. After trypsinization, 300 μl 4-mg/ml trypsin inhibitor (soybean; Boehringer Mannheim GmbH) in 0.05 M KH_2_PO_4_ with 0.005 M EDTA (pH 6.1) was added, and the samples were serially diluted in TG buffer and plated on 4% horse blood agar for counting of CFU.

### Coagulation experiments with and without bacteria.

Coagulation of normal plasma without anticoagulant was performed in microwell plates (Nunclon Delta Surface; Nunc A/S), and the absorbance at 490 nm was measured over time during incubation at 37°C. To all samples, 300 μg of human fibrinogen (Sigma) was added to amplify the signal. The treatments used are given in Table S1 in the supplemental material. The plasminogen was from Technoclone GmbH. The anti-FVII antibody was from Sanquin (murine, clone CLB VII-1 [characterized in reference 25]), and the control antibody was from Statens Seruminstitut (murine, HYB 332-01). The FXII and PK inhibitor H–d–Pro–Phe–Arg–CMK was from Bachem AG.

### Enzyme assays.

For assessment of FXII/PK activation in pure solutions, microwells (MaxiSorp; Nunc A/S) were incubated overnight at 4°C with 200 μl 10-μg/ml human FXII (Kordia) in 1.69 g/liter Na_2_CO_3_ with 2.94 g/liter NaHCO_3_ (pH 9.6) or with this buffer only. After being washed with phosphate-buffered saline (PBS), 200 μl NaCl solutions (38 mM or 145 mM for Fig. 3b, or the range of ionic strengths given in Fig. 3a) were added with or without 5 μg/ml (final concentration) human PK (Enzyme Research Laboratories), and samples were incubated at 37°C for 1 h. The wells were washed with PBS and incubated with 200 μl of the chromogenic FXII/PK substrate S-2302 (Chromogenix) for 2 h at room temperature, and the absorbance at 405 nm was measured. (For Fig. 3a, samples without PK or with no proenzyme were used as negative controls.)

For assessment of FXII/PK activity at the bacterial surface, G45 bacteria were washed and diluted to 2 × 10^9^ CFU/ml in PBS. Three hundred microliters of this suspension was centrifuged (13,000 RCF), the pellet was resuspended in 900 μl 38 mM NaCl or 145 mM NaCl, and 100 μl of normal citrated plasma was added. Alternatively the pellet was resuspended in 1,000 μl 38 mM or 145 mM NaCl. The suspensions were incubated at 37°C for 1 h under rotation. The bacteria were then washed in PBS, resuspended in 100 μl of the chromogenic FXII/PK substrate S-2302, and incubated for 30 min at room temperature. The samples were centrifuged, and the absorbance at 405 nm of the supernatant was measured in microwells. Plasma from eight different donors (four of each sex) was used.

### Electron microscopy.

For visualization of saliva-plasma clots with streptococci, G45 bacteria were harvested in the mid-exponential phase, washed, and diluted to 2 × 10^9^ CFU/ml in PBS. A 150-μl bacterial suspension was pelleted by centrifugation, and the pellet was resuspended in 450 μl saliva with the addition of 50 μl plasminogen-depleted plasma and 7.5 μl of 0.2 M CaCl_2_ and incubated at 37°C for 1 h. For thrombin-induced clotting, PBS with the addition of 0.3 μl of 3.3 mg/ml thrombin (Innovative Research) was substituted for the saliva. Samples without bacteria were prepared as described above, but with the omission of bacteria.

For the dissolution of clots, samples were prepared as described above for saliva-plasma clots, but with a 10-μl bacterial suspension, with or without the addition of 10 μl of 1 mg/ml plasminogen. The samples were incubated at 37°C for the times indicated in the figure legend. Specimens were fixed in 2.5% glutaraldehyde in 0.15 M sodium cacodylate at pH 7.4 (cacodylate buffer) for 30 min at room temperature, washed with cacodylate buffer, and dehydrated with an ascending ethanol series from 50% (vol/vol) to absolute ethanol (10 min per step). The samples were then subjected to critical-point drying in carbon dioxide, with absolute ethanol as the intermediate solvent, mounted on aluminum holders, and finally sputtered with 30-nm palladium-gold particles. Specimens were examined in a high-resolution FEI scanning electron microscope (FEG) at the Core Facility for Integrated Microscopy (CFIM) at Panum Institute, University of Copenhagen. At least 50 independent image profiles were visualized and analyzed in each case, and representative fields were chosen to prepare the figures.

For visualization of intrinsic pathway proenzymes, FXII and PK were incubated separately at a final concentration of 10 μg/ml in 38 or 145 mM NaCl solutions for 1 h at 37°C. The samples were subjected to negative staining with 0.75% uranyl formate and transmission electron microscopy as described in reference 26. Specimens were visualized in a Philips/FEI CM100 twin electron microscope equipped with an LaB6 filament and a side-mounted Olympus Megaview-2 camera.

### Characterization of clots and measurement of streptokinase and intracellular proteins.

Three hundred microliters of 2 × 10^9^ CFU/ml G45 bacteria in PBS was pelleted by centrifugation and resuspended in 900 μl saliva. To this was added 100 μl saliva or 100 μl plasminogen-depleted plasma and 15 μl 0.2 M CaCl_2_ to recalcify the citrated plasma. Samples were incubated for 1 h at 37°C under rotation. Coagula and bacteria were pelleted by centrifugation and washed four times in PBS, and pellets and supernatants were stored at −80°C until analysis. Alternatively, samples were not fractionated into pellets and supernatants. Experiments with only saliva and plasma were performed as described above, but with the omission of bacteria. The samples were analyzed by mass spectrometry (MS) as described below. For the assessment of fibrin formation, the following peptides were measured: fibrinopeptide A (ADSGEGDFLAEGGGVR), fibrinopeptide B (Q[−17.0]GVNDNEEGFFSAR), and peptides located centrally within the α (QHLPLIK, NSLFEYQK, GSESGIFTNTK), β (GSWYSMR, EDGGGWWYNR, MGPTELLIEMEDWK) and γ (LDGSVDFK, DTVQIHDITGK, YLQEIYNSNNQK) chains of fibrinogen.

### LC-MS/MS analysis.

Protein samples were digested and prepared for mass spectrometry (MS) as described previously (27). Peptides were analyzed by liquid chromatography-tandem mass spectrometry (LC-MS/MS) using Easy-nLC (nano-liquid chromatography) systems connected to either a Q-Exactive Plus or a TSQ Quantiva mass spectrometer equipped with EasySpray electrospray ion sources. Peptide separation was performed on EasySpray ES800 or ES802 columns (all Thermo Scientific).

### DDA MS analysis.

MS/MS spectra were acquired in data-dependent analysis (DDA) mode, selecting the 15 most abundant precursor ions (*m*/*z* of 400 to 1,600 with 70,000 resolving power at *m*/*z* of 200) for fragmentation (17,500 resolving power at *m*/*z* of 200) per cycle. The raw data were searched with the Trans-Proteomic Pipeline (28) against the PTHR9.0_human database (29) and quantified using OpenMS (30)—all within the iPortal/OpenBIS workflow management (31, 32). Final data analysis was done with DDB (33, 34).

### SRM MS.

Selected reaction monitoring (SRM) assays were either obtained from DDB (20, 27, 35) or developed (for fibrinopeptides A and B and streptokinase) as previously described (36). SRM data were collected using widths of 0.7 full width at half-maximum (FWHM) in both quadrupoles and 5 ms of dwell time. Data analysis was performed with the Skyline software (37) and Prism 6 (GraphPad Software Inc.).

### Clearance of streptococci from epithelial cells.

Primary human epidermal keratinocytes (Cascade Biologics) were precultured in 12-well plates (Falcon, Corning Inc.) with KGM-Gold from Lonza, supplemented with KGM SingleQuots (Portsmouth NH) and 100 ng/ml epidermal growth factor (EGF) (Peprotech) for 72 h. The cells were then cultured in KGM without EGF and insulin for 48 h, incubated with 20% saliva in KGM-Gold without antibiotics for 24 h, and washed in PBS prior to the addition of bacteria. A total of 2 × 10^10^ CFU/ml G45 bacteria were incubated with 0.2 mg/ml fluorescein isothiocyanate (FITC) in PBS for 30 min on ice and washed, and 10 μl of bacterial suspension was mixed with the following: 300 μl PBS with 3 μl chloramphenicol (1-mg/ml stock); 300 μl saliva with 3 μl chloramphenicol; 270 μl saliva, 30 μl normal autologous plasma (without anticoagulant), and 3 μl chloramphenicol; 270 μl saliva, 30 μl plasma, 3 μl chloramphenicol, and 17 IU/ml (final concentration) heparin (preincubated with the plasma for 10 min on ice); or 270 μl saliva with 30 μl plasma. The mixtures were placed on top of the keratinocytes, and the plates were incubated at 37°C for 1 h under gentle rotation. An inoculation loop (1-μl size) was pulled one turn along the rim of each well, and the wells were washed with PBS and incubated with TrypLE select (Gibco, Denmark). After trypsinization, the cells were transferred to a black 96-well microtiter plate (Nunc, Denmark). The fluorescence was measured at 535 nm (fluorescein channel) with a Victor 1420 multilabel reader (PerkinElmer).

## RESULTS

### Initial observations.

**FIG 1:**
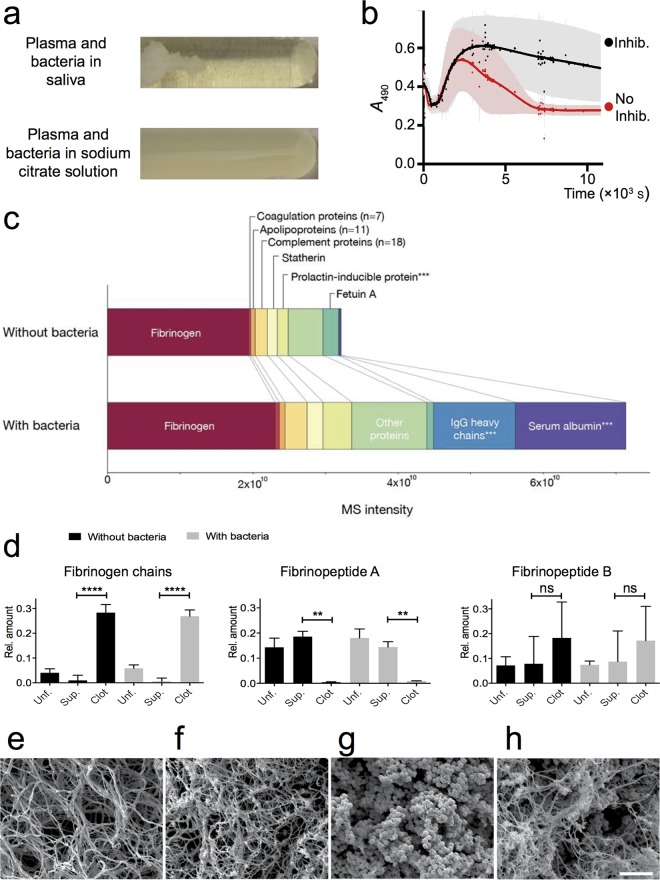
(a) Visualization of clotting and entrapment. Plasma and G45 bacteria were added to saliva or sodium citrate solution and incubated at 37°C. Chloramphenicol was used to prevent protein synthesis. (b) Clot dissolution. Plasma and G45 bacteria were added to saliva with (Inhib. [black]) or without (No Inhib. [red]) the protein synthesis inhibitor chloramphenicol. Clotting was monitored as the change in absorbance over time during incubation at 37°C. Measurements were performed every 6 s, and no curves have been fitted. Shown are the mean and 95% confidence interval (CI) for six replicates in two blocks, with different bacterial cultures for replicates and different donors of saliva and plasma for blocks. (c) Protein content of clots. Plasminogen-depleted plasma was incubated with saliva with or without G45 bacteria. The samples were then fractionated by centrifugation, and the pellets were washed. A total of 170 human proteins were identified and quantified by shotgun LC-MS/MS. The bar charts show the total human protein signal and the abundances of selected proteins and protein groups (for details, see Table S1 in the supplemental material) in the clots. Results are represented as median values from three replicates with different bacterial cultures and different donors of saliva. A group marked with *** contains proteins with significant differences (>2-fold change) (*P* < 0.001, multiple *t* test) between samples with and without bacteria: Ig gamma-1 chain C region, Ig gamma-2 chain C region, Ig lambda-1 chain C region, prolactin-inducible protein, and serum albumin. (d) Spatial separation of fibrinopeptides and fibrinogen chains. Plasminogen-depleted plasma was incubated with saliva with or without G45 bacteria. Some samples were fractionated by centrifugation. The pellet and supernatant (Sup.) as well as unfractionated (Unf.) samples were homogenized and trypsin digested. Fibrinopeptides A and B, as well as nine tryptic peptides located centrally within the fibrinogen α, β, and γ chains, were quantified by targeted proteomics (SRM MS). The acquired sample peptide intensities are plotted as fractions of the total peptide intensity within the sample group (with or without bacteria). Shown are the mean and standard deviation (SD) from three replicates with different bacterial cultures and different donors of saliva. The level of statistical significance is indicated by **** for *P* < 0.0001 and ** for *P* < 0.01, with “ns” indicating “not significant,” as assessed with the Welch corrected *t* tests. (e to h) Electron microscopy of clots with and without streptococci. (e) Saliva-plasma clot without bacteria. (f) Plasma clot without bacteria, formed by incubation of plasma with thrombin. (g) Saliva-plasma clot with bacteria. (h) Plasma clot with bacteria, formed by incubation with thrombin. The scale bar represents 5 μm.

This study originated from a simple observation. When streptococci isolated from an episode of pharyngitis were incubated with a mixture of saliva, plasma, and the protein synthesis inhibitor chloramphenicol (to inhibit the production of streptokinase), the originally turbid suspension separated into clots surrounded by a clear fluid that appeared depleted of bacteria (Fig. 1a). Further experiments indicated that in the absence of chloramphenicol, the bacteria were able to reverse the clotting (Fig. 1b).

In the following text, we characterize these phenomena, investigate the molecular mechanisms involved, and address their potential functions in streptococcal pharyngitis.

### Clots consist of fibrin and other plasma and saliva proteins.

Saliva is known to induce the clotting of blood and plasma (25, 38, 39), but the phenomenon has not been characterized under conditions of pharyngitis. We therefore used mass spectrometry to investigate the composition of clots formed in mixtures of 10% plasma (plasminogen depleted to prevent clot dissolution) in saliva. In the absence of bacteria, the bulk of the MS signal represented fibrinogen (Fig. 1c), other notable constituents being complement proteins and prolactin-inducible protein (Fig. 1c). When streptococci were added, there were large increases in IgG and serum albumin (Fig. 1c), both of which are known to bind to the bacteria used (40, 41). The full results are reported in Table S2 in the supplemental material.

To investigate whether the fibrinogen signal represented fibrin, we measured fibrinopeptides A and B as well as peptides located centrally within the α, β, and γ chains of fibrinogen. In both the presence and absence of streptococci, the fibrinogen chains were enriched in the clots, whereas fibrinopeptide A was depleted, and fibrinopeptide B showed an intermediate pattern (Fig. 1d), which is consistent with the formation of fibrin. Figure 1e through h show electron microscopy images of saliva-plasma clots in the absence (Fig. 1e) or presence (Fig. 1g) of streptococci. Clots formed with added thrombin (no saliva) are included for comparison (Fig. 1f and h).

### Identification of candidate mechanisms of coagulation system activation.

To identify candidate mechanisms, we performed experiments in which the progression of coagulation over time was monitored by measurements of the absorbance at 490 nm. Except for negative controls with only saliva, normal plasma (without anticoagulant) at a final concentration of 10% was used in all treatments. In order to facilitate the comparison among treatments and the pooling of data, the material for these experiments was withdrawn from two donors who had the same Ca^2+^ concentrations in both protein-depleted saliva and protein-depleted plasma (not shown).

**FIG 2:**
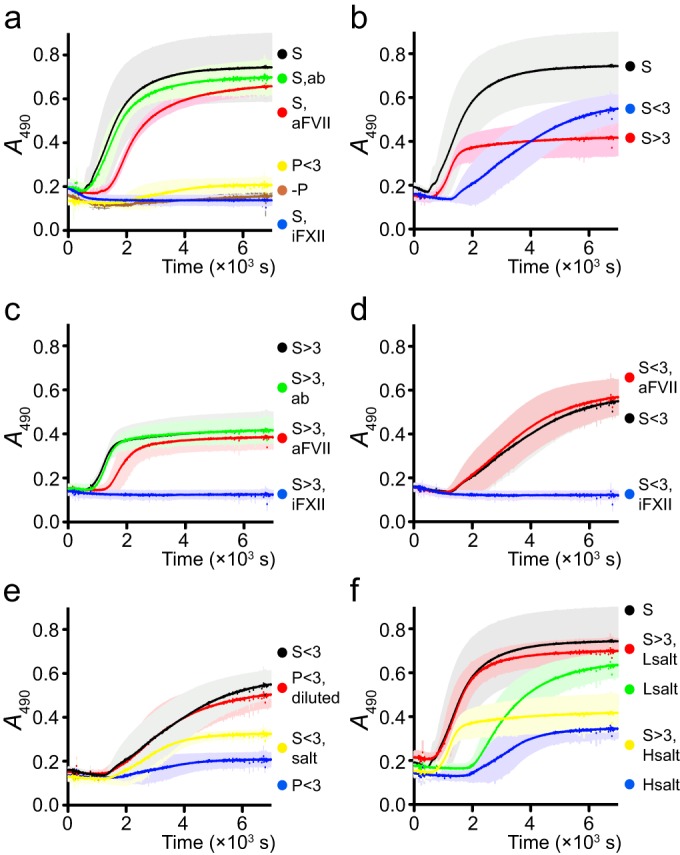
Clotting was monitored as the change in absorbance at 490 nm over time during incubation at 37°C. Measurements were performed every 6 s, and no curves have been fitted. Some results are given in several panels for comparison. Shown are the mean and 95% CI for six replicates in two blocks with different donors of saliva and plasma for blocks. (a) Pathways of coagulation. Plasma was diluted to a final concentration of 10% in saliva (S [black]), saliva with an inhibiting antibody against FVII (S, aFVII [red]), saliva with a control antibody (S, ab [green]), saliva with an inhibitor of FXII/PK (S, iFXII [blue]), or plasma that had been passed through a 3-kDa filter (P<3 [yellow]). Saliva with no plasma added was used as a negative control **(−**P [brown]). (b) Fractions of saliva. Plasma was diluted to a final concentration of 10% in saliva (S [black]), saliva passed through a 3-kDa filter (S<3 [blue]), or the fraction of saliva recovered with a 3-kDa filter (i.e., the protein fraction) that had been washed and diluted to the original concentration in an NaCl-CaCl_2_ solution with the same ionic strength as plasma, and the same calcium concentration as protein-depleted saliva and plasma (S>3 [red]). (c) Protein fraction. Plasma was diluted to a final concentration of 10% in the fraction of saliva recovered with a 3-kDa filter (i.e., the protein fraction) that had been washed and diluted to the original concentration in an NaCl-CaCl_2_ solution with the same ionic strength as plasma and the same calcium concentration as protein-depleted saliva and plasma. To this mixture, the following was added: nothing (S>3 [black]), control antibody (S>3, ab [green]), anti-FVII antibody (S>3, aFVII [red]), or inhibitor of FXII/PK (S>3, iFXII [blue]). (d) Non-protein fraction. Plasma was diluted to a final concentration of 10% in saliva passed through a 3-kDa filter. To this mixture, the following was added: nothing (S<3 [black]), anti-FVII antibody (S<3, aFVII [red]), or an inhibitor of FXII/PK (S<3, iFXII [blue]). (e) Ionic strength. Plasma was diluted to a final concentration of 10% in saliva passed through a 3-kDa filter (S<3 [black]), plasma passed through a 3-kDa filter (P<3 [blue]), plasma passed through a 3-kDa filter and diluted to an ionic strength similar to that of saliva while holding the calcium concentration constant (P<3, diluted [red]), or saliva passed through a 3-kDa filter with NaCl added to a final ionic strength similar to that of plasma (S<3, salt [yellow]). (f) Combining components. Plasma was diluted to a final concentration of 10% in saliva (S[black]), an NaCl-CaCl_2_ solution with the same ionic strength as plasma and the same calcium concentration as protein-depleted saliva and plasma (Hsalt [blue]), an NaCl-CaCl_2_ solution with the same ionic strength as saliva and the same calcium concentration as protein-depleted saliva and plasma (Lsalt [green]), the fraction of saliva recovered with a 3-kDa filter that had been washed and diluted to the original concentration in an NaCl-CaCl_2_ solution with the same ionic strength as plasma and the same calcium concentration as protein-depleted saliva and plasma (S>3, Hsalt [yellow]), or the fraction of saliva recovered with a 3-kDa filter that had been washed and diluted to the original concentration in an NaCl-CaCl_2_ solution with the same ionic strength as saliva and the same calcium concentration as protein-depleted saliva and plasma (S>3, Lsalt [red]).

The coagulation system has two interconnected branches. The extrinsic pathway is initiated by tissue factor (TF) and factor VII (FVII), while the intrinsic pathway is initiated by the reciprocal activation of factor XII (FXII) and plasma kallikrein (PK). It is common to use “FXIIa” to denote the activated form of FXII and “PPK” for the nonactivated form of PK, but here we will consistently use “FXII” and “PK” for readability. In saliva-plasma mixtures, inhibition of the extrinsic pathway with an antibody against FVII delayed the onset of coagulation compared to samples without antibody or with a control antibody, but there was no significant effect on the final level of absorbance (Fig. 2a), as determined by the 95% confidence intervals. Inhibition of the intrinsic pathway with a synthetic FXII/PK inhibitor abolished clotting (Fig. 2a). One-tenth of the concentration of this inhibitor almost completely inhibited clotting as well (not shown). As a control, protein-depleted plasma was used. Whole plasma was passed through a centrifugal filter with a 3-kDa cutoff (P<3) and then mixed with 10% (final concentration) whole plasma, in order to attain the same concentration of coagulation proenzymes as in other treatments without introducing any potentially pro- or anticoagulant factors. These samples, as well as negative controls with only saliva (no plasma added), showed little or no clotting (Fig. 2a). The results indicate that saliva induces clotting and suggest that it proceeds through both pathways of coagulation, with the two pathways exerting different effects.

To tease these effects apart, we separated saliva into a protein fraction and a protein-depleted fraction, using centrifugal filters with a 3-kDa cutoff, and the effect of these fractions on the different pathways of coagulation was investigated. The protein fraction (S>3) induced clotting as rapidly as whole saliva, but the final level of absorbance was reduced (Fig. 2b). The addition of an anti-FVII antibody delayed clotting (Fig. 2c), showing that the protein fraction activates the extrinsic pathway. Clotting in the protein-depleted fraction (S<3) was slower (Fig. 2b). It was not affected by the anti-FVII antibody (Fig. 2d), which indicates that clotting in this fraction does not involve the extrinsic pathway, but relies on the intrinsic pathway. The FXII/PK inhibitor abolished clotting in both fractions (Fig. 2c and d).

Next, the factor in the protein-depleted saliva (S<3) affecting the intrinsic pathway was investigated. Since, as previously mentioned, the Ca^2+^ concentrations were the same in protein-depleted saliva (S<3) and protein-depleted plasma (P<3) from both donors, the difference in clotting between plasma mixed with P<3 and with S<3 seen in Fig. 2e was not due to a difference in calcium levels. However, coagulation is affected by ionic strength (42), and saliva has much lower ionic strength than plasma. (The rationale for the choice of ionic strengths is given in Materials and Methods.) To investigate whether the difference in clotting was due to a difference in ionic strength, protein-depleted plasma was diluted to an ionic strength similar to that of saliva (P<3, diluted), without changing the calcium concentration. The clotting characteristics of plasma in this diluted protein-depleted plasma were very similar to those in protein-depleted saliva (S<3 [Fig. 2e]). Similarly, when protein-depleted saliva was supplemented with NaCl to increase its ionic strength to the level of plasma (S<3, salt), its coagulant properties became more similar to those of protein-depleted plasma (P<3 [Fig. 2e]). To further investigate this effect, NaCl solutions with the same ionic strength as saliva (Lsalt [38 mM]) or plasma (Hsalt [145 mM]) supplemented with CaCl_2_ were used. At both ionic strengths, clotting was slow, but the final level of absorbance was higher at low ionic strength (Fig. 2f), consistent with a positive effect of low ionic strength on the intrinsic pathway.

The results so far indicate that the effect of saliva on clotting has two components. Salivary protein induces the extrinsic pathway, the low ionic strength induces or potentiates the intrinsic pathway, and the total effect of whole saliva is due to the combination of these two components. To investigate this, the protein fraction of saliva was washed and diluted to the original concentration in the NaCl-CaCl_2_ solution with the same ionic strength as saliva (Lsalt). This reconstituted saliva had clotting characteristics similar to those of whole saliva (Fig. 2f).

### The low ionic strength of saliva activates the intrinsic pathway of coagulation.

**FIG 3:**
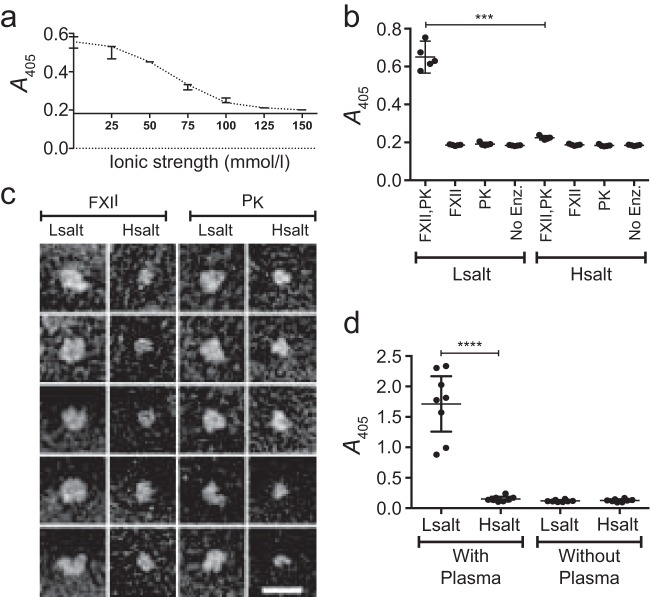
(A) Activation of FXII as a function of ionic strength. FXII was immobilized in microwells and incubated with PK in NaCl solutions with different ionic strengths. The wells were then washed and incubated with a chromogenic substrate for FXII/PK. The enzymatic activity was assessed as absorbance at 405 nm. The *x* axis is drawn at the absorbance of negative controls. Shown are the median and range from three replicates. (b) Activation of FXII in a pure system. The following combinations of proenzymes were incubated in microwells: immobilized FXII and soluble PK (FXII, PK), immobilized FXII only (FXII), soluble PK only (PK), or no proenzyme (No Enz) as a negative control. All combinations of proenzymes were incubated in NaCl solutions with the same ionic strength as saliva (Lsalt) and plasma (Hsalt), respectively, and the enzymatic activity was assessed as in panel a. Shown are the individual values, mean, and 95% CI for five replicates. The level of statistical significance is indicated by *** for *P* = 0.0001, as assessed with the Welch corrected *t* test. (c) Visualization of FXII and PK at different ionic strengths. FXII and PK were incubated (separately) in NaCl solutions with the same ionic strength as saliva (Lsalt) and plasma (Hsalt), respectively. The samples were then subjected to negative staining and electron microscopy. The scale bar represents 25 nm. (d) FXII/PK activation at the bacterial surface. G45 bacteria were incubated with plasma in NaCl solutions with the same ionic strength as saliva (Lsalt) or plasma (Hsalt). Samples without plasma were used as negative controls. After incubation, the bacteria were washed and resuspended in a chromogenic substrate for FXII/PK, and the enzymatic activity was assessed as absorbance at 405 nm. Shown are the individual values, mean, and 95% CI from eight replicates with separate bacterial cultures and different donors of plasma. The level of statistical significance is indicated as **** for *P* < 0.0001, as assessed with the Welch corrected *t* test.

To test the findings from the clotting experiments, we investigated the effect of ionic strength on the initiating (pro)enzymes of the intrinsic pathway. FXII was immobilized on a plastic surface and incubated with NaCl solutions of different ionic strengths, with or without PK. The surface was then washed, to remove any PK and equilibrate the ionic strength, and incubated with a synthetic FXII/PK substrate. The results showed that the activation of FXII was dependent on PK and was greater at the low ionic strength corresponding to saliva than at the high ionic strength corresponding to plasma (Fig. 3a and b). (See Materials and Methods for the bases of these ionic strengths.) In addition, using electron microscopy, FXII and PK were visualized at the ionic strengths of saliva and plasma, respectively, and the images suggest that both proteins adopt a more open conformation at low ionic strength (Fig. 3c).

### Low ionic strength activates intrinsic pathway proenzymes at the bacterial surface.

Components of the intrinsic pathway interact with several bacteria, including streptococci (43–45). To investigate the effect of ionic strength on this interaction, streptococci were incubated with 10% plasma in NaCl solutions with the same ionic strength as saliva or plasma. The bacteria were washed to equilibrate the ionic strengths, and the activity of FXII and PK associated with the bacterial surface was assessed with a synthetic substrate. (FXII and PK bind to surface proteins of the bacteria used [43].) At low ionic strength, the enzymes were activated, whereas at high ionic strength, activity was low and similar to that of negative controls without plasma (Fig. 3d).

### Streptococci are entrapped in the clots but escape by activating plasminogen.

In Fig. 1a, the fluid surrounding the clot is clear, despite the fact that the nonclotted control treatment is turbid with bacteria, and the graphs in Fig. 1b show reversion of clotting in the absence of the protein synthesis inhibitor chloramphenicol. Together these observations suggest that streptococci are entrapped in the clots and escape by a fibrinolytic mechanism. As streptococci associated with pharyngitis (groups A, C, and G, represented by Streptococcus pyogenes and S. dysgalactiae subsp. equisimilis) are known to produce streptokinase (46, 47), which activates host plasminogen into fibrinolytic plasmin, this would be an obvious candidate mechanism for any escape.

**FIG 4:**
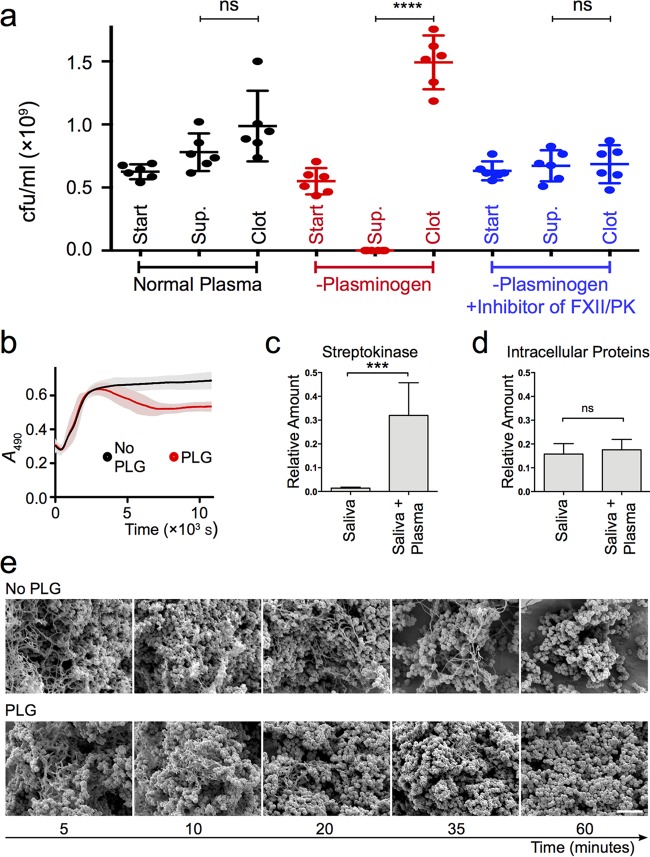
(a) Entrapment with and without plasminogen. G45 bacteria were incubated in saliva with the addition of normal plasma (black), plasminogen-depleted plasma (red), or plasminogen-depleted plasma with an inhibitor of FXII and PK (blue). Each sample was split into three aliquots. One was withdrawn prior to incubation (start). A second was withdrawn from the fluid phase after incubation (Sup.), and the third was the remaining material containing any clots (Clot). All aliquots were trypsinated, and the number of CFU was determined. Shown are individual values, mean, and 95% CI for six replicates in two blocks with different bacterial cultures for replicates and different donors of saliva and normal plasma for blocks. The level of statistical significance is indicated by **** for *P* < 0.0001, with “ns” indicating “not significant,” as assessed with the Welch corrected *t* tests. (b) Clotting with and without plasminogen. G45 bacteria were incubated in saliva with the addition of plasminogen-depleted plasma with (PLG [red]) or without (No PLG [black]) exogenous plasminogen. Clotting was monitored as the change in absorbance at 490 nm over time during incubation at 37°C. Measurements were performed every 6 s, and no curves have been fitted. Shown are the mean and 95% CI for six replicates in two blocks, with different bacterial cultures for replicates and different donors of saliva for blocks. (c and d) Production of streptokinase. G45 bacteria were incubated in saliva with or without plasminogen-depleted plasma, and the total samples were homogenized. Peptides derived from streptokinase (c) and 15 intracellular proteins (d) were quantified with SRM MS. The acquired sample peptide intensities are plotted as fractions of the total peptide intensity. Shown are the mean and SD from three replicates with different bacterial cultures and different donors of saliva (***, *P* < 0.001, and ns, not significant, as assessed with Student's *t* test). (e) Electron microscopy of dissolving saliva-plasma clots. Streptococci were incubated in mixtures of saliva and plasminogen-depleted plasma without (upper row) or with (lower row) added plasminogen. From left to right are samples incubated for 5, 10, 20, 35, and 60 min, respectively. The scale bar represents 5 μm.

To address these issues, we incubated streptococci in mixtures of saliva and plasma, withdrawing material prior to incubation (“Start” in Fig. 4a), as well as from the fluid phase (Sup.) and remaining material (Clot) after incubation. The material was trypsinated to dissolve any clots, and the number of CFU was determined.

In normal plasma, where plasminogen is available for bacterial activation, CFU counts were similar in all fractions (Fig. 4a). In contrast, with plasminogen-depleted plasma, almost no CFU were found in the Sup. fraction (Fig. 4a), whereas CFU counts were elevated in the Clot fraction (Fig. 4a). These results support the conjecture that the bacteria are entrapped in the clots but are able to escape by activating host plasminogen. When an inhibitor of the intrinsic pathway was added to the plasminogen-depleted plasma, no entrapment was observed (Fig. 4a).

The mechanism of escape by plasminogen activation was further established with clotting experiments in the presence and absence of plasminogen (Fig. 4b) and the measurement of streptokinase production in response to plasma (Fig. 4c and d). The electron microscopy images in Fig. 4e show time series of streptococci in saliva-plasma clots in the absence (upper row) or presence (lower row) of plasminogen.

### Both pathogenic and commensal streptococcal strains are entrapped, but only the pathogenic strains escape the clot.

**FIG 5:**
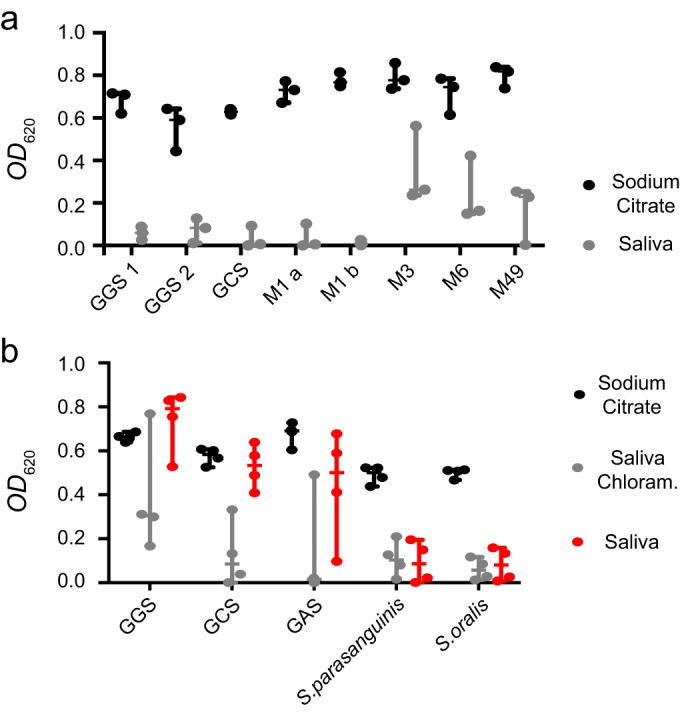
(A) Entrapment of different groups and serotypes. A range of streptococcal strains were incubated with plasma and chloramphenicol in either sodium citrate solution (the left-hand bar in each pair [black]) or saliva (the right-hand bar in each pair [gray]). The optical density (OD_620_) of the fluid phase was measured. Strains are denoted according to group (for GGS and GCS) and serotype (for GAS). Shown are the individual values, median, and 95% CI from three replicates with different bacterial cultures and different donors of saliva and plasma. (b) Escape of pathogenic and commensal streptococci. Streptococci were incubated with plasma in either sodium citrate solution (black) or in saliva with (gray) or without (red) chloramphenicol (Chloram.). The optical density (OD_620_) of the fluid phase was measured. Strains are denoted according to group or species. Shown are individual values, median, and 95% CI from four replicates with different bacterial cultures and different donors of saliva and plasma.

To address the generality of entrapment among group A, C, and G streptococci, we incubated a number of strains of different groups and serotypes in mixtures of saliva and plasma with the addition of chloramphenicol, and measured the optical density (OD) of the fluid phase as a proxy for the number of bacteria. Samples with sodium citrate solution instead of saliva were used as coagulation-free controls. For all strains, the OD was lower in the fluid from saliva samples than that from sodium citrate control samples (Fig. 5a), indicating that entrapment is not strain specific.

To assess the generality of bacterial escape among pathogenic and commensal streptococci, we used three strains of pathogenic streptococci (S. pyogenes and S. dysgalactiae subsp. equisimilis, comprising groups A, C, and G) as well as two strains of commensal streptococci (S. parasanguinis and S. oralis). The bacteria were incubated in mixtures of saliva and plasma with or without chloramphenicol, and the OD was measured as a proxy for bacterial number, as described above. There was considerable variation among replicates, but there was a general tendency that both pathogenic and commensal streptococci were entrapped, whereas only the pathogenic strains were able to escape the clot (Fig. 5b). Consistent with this pattern, BLAST searches revealed no close homologues of streptokinase in reference genomes of the commensal species.

### Saliva-induced clotting may clear streptococci from the epithelial surface.

**FIG 6:**
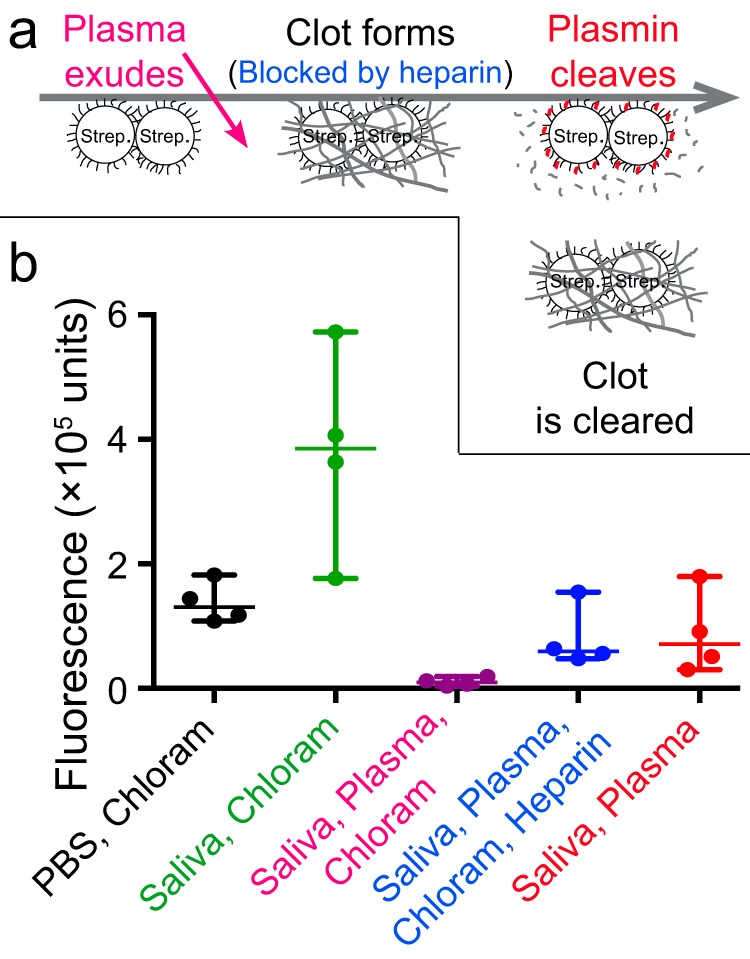
(a) Hypothetical model of streptococcal pharyngitis. Plasma exudes into the airway lumen and mixes with saliva. The coagulation system is activated, and the bacteria are entrapped in clots. In the absence of a bacterial response, the clots and bacteria are cleared by swallowing, whereas the bacterial response results in plasmin activity, clot dissolution, and counteraction of clearance. The gray line represents the epithelium as well as the time axis. (b) Test of the hypothesis. Fluorescence-labeled G45 bacteria with or without the addition of the protein synthesis inhibitor chloramphenicol were incubated at an epithelial cell surface covered in PBS, saliva, or a saliva-plasma mixture, with or without heparin to inhibit coagulation. The cells were washed, and the fluorescence associated with them was measured. Shown are individual values, median, and 95% CI for four replicates in two blocks with different bacterial cultures for replicates and different donors of saliva and plasma for blocks.

In streptococcal pharyngitis or tonsillitis, plasma exudes into the upper airways. Based on the results reported above, we hypothesized that when plasma mixes with saliva in the lumen or on the tonsillar surface, it will coagulate and entrap the streptococci in clots. We speculated that such clotting may contribute to mechanical clearance of the bacteria through swallowing and that the escape mechanism described above would allow the bacteria to remain at the epithelium (Fig. 6a).

To test the viability of this hypothesis, fluorescence-labeled streptococci were incubated on an epithelial cell surface covered by saliva, and the fluorescence was used to assess the ability of the bacteria to avoid clearance. We used FITC as a label in order to make the fluorescence of each sample represent the mechanical clearance of the bacteria, rather than transcription, proliferation, or killing, which may otherwise have confounded the results. The material was trypsinated prior to measurement to prevent the fibrin clots from interfering with fluorescence readings.

As seen in Fig. 6b, the addition of plasma to chloramphenicol-treated bacteria in saliva resulted in a large reduction in fluorescence. This reduction was attenuated by the addition of heparin and to a similar extent by the omission of chloramphenicol (Fig. 6b). These results support the hypothesis that clotting may contribute to the clearance of bacteria from the epithelial surface and that the bacteria are able to counteract this clearance mechanism.

## DISCUSSION

Here we report that saliva activates the coagulation system, including FXII of the intrinsic pathway, that saliva-induced clotting entraps streptococci, and that pathogenic streptococci are able to escape entrapment by activating fibrinolysis. We also suggest a hypothesis as to the functions of these phenomena for the host and pathogen, respectively, in streptococcal pharyngitis. A given mechanism may have more than one function, and the clots could, for example, also limit bacterial spread in the airways, protect damaged epithelium, and/or inhibit the diffusion of bacterial products such as exotoxins.

The mechanisms by which saliva induces coagulation have been previously addressed (25). That study reported that saliva contains TF that initiates coagulation via the extrinsic pathway but found no effect on the intrinsic pathway as assessed with an anti-FXII antibody (25). Our results on extrinsic pathway activation are in line with this previous study, while the finding that the intrinsic pathway is activated by low ionic strength may seem contradictory. However, the study mentioned above, addressing the issue of wound licking, used a high concentration of plasma, which results in a high ionic strength. We, in contrast, focusing on an inflammatory exudate in the upper airways, used a lower concentration of plasma, yielding a lower ionic strength.

The finding that both pathways of coagulation are involved raises the question of their respective roles. Our results suggest that in the saliva-plasma mixture, the extrinsic pathway initiates the coagulation process, whereas the intrinsic pathway is required for the full development of the fibrin clot. This is similar to the division of labor between the pathways described for coagulation induced by microparticles (48).

The molecular mechanisms by which streptococci interfere with host defenses have been the subject of extensive research, and accordingly, the action of streptokinase on the fibrinolytic system is well known. However, the heavy focus on the clinically important but relatively rare invasive infections has left the adaptive significance of streptococcus-host interactions largely obscure. In line with recent theoretical work on streptococcal virulence (14), however, the results we present here suggest the possibility that streptokinase may be an adaptation for pharyngitis.

Much work remains to be done, especially characterizing noninvasive host-pathogen interactions *in vivo*. At present, there is a lack of suitable animal models, but technological advances in, for example, proteomics should facilitate future studies in human patients with superficial infections. Such studies may contribute to a better understanding of streptococcal adaptations by elucidating the mechanisms and functions of streptococcus-host interactions in the settings that constitute the vast majority of the infections.

## Supplementary Material

Supplemental material
