# Supplementary material for: Saliva-Induced Clotting Captures Streptococci: Novel Roles for Coagulation and Fibrinolysis in Host Defense and Immune Evasion
Source: Infect Immun. 2016 Sep 19;84(10):2813–23. doi: 10.1128/IAI.00307-16 (PMC5038080; doi:10.1128/IAI.00307-16)
Supplement: Supplemental material [file supp_84_10_2813__index.html]

Saliva-Induced Clotting Captures Streptococci: Novel Roles for Coagulation and Fibrinolysis in Host Defense and Immune Evasion — Supplemental material 

# Saliva-Induced Clotting Captures Streptococci: Novel Roles for Coagulation and Fibrinolysis in Host Defense and Immune Evasion

## Supplemental material

- Supplemental file 1 -

  Table S1. Coagulation experiments with and without bacteria. Table S2. List of proteins and protein chains quantified in saliva-plasma clots with and without bacteria using shotgun MS.

  PDF, 523K
