# Supplementary material for: Saliva-Induced Clotting Captures Streptococci: Novel Roles for Coagulation and Fibrinolysis in Host Defense and Immune Evasion
Source: Infect Immun. 2016 Sep 19;84(10):2813–23. doi: 10.1128/IAI.00307-16 (PMC5038080; doi:10.1128/IAI.00307-16)
Supplement: Supplemental material [file IAI.00307-16_zii999091834so1.pdf]

Supplementary material

Legends to supplementary tables

Supplementary table S1

Coagulation experiments with and without bacteria. Each row represents a treatment. H-D-Pro-Phe-Arg-CMK is an inhibitor of FXII and PK. P<3 is the fraction of plasma smaller than 3 kDa. S<3 is the fraction of saliva smaller than 3 kDa. S>3 is the fraction of saliva larger than 3 kDa. Fractions are the flow-through and retentate, respectively, from centrifugal filters with a 3 kDa cut-off.

Supplementary table S2

List of proteins and protein chains quantified in saliva-plasma clots with and without bacteria using shotgun MS. The intensity values are from three biological replicates per sample-group. The denoted protein groups are the same as used in *fig. 1 c*. The ignored group contains protein chains of which intensity values were omitted in *fig. 1 c*. The proteins were identified with the Trans Proteomic Pipeline software environment using 1% False Discovery Rate (FDR), and Label-free quantification values were determined by OpenMS. Multiple t-test (one by row) of replicate intensity values calculated with the software GraphPad Prism 6.0 assuming that all rows were sampled from populations with equal variance and the Q-value (FDR) set to 1%.

Supplementary tables

Supplementary table S1

Coagulation experiments with and without bacteria.

**Coagulation experiments with bacteria**				
180 µl saliva	20 µl plasma	4×10^{7} cfu G45 bacteria		
180 µl saliva	20 µl plasma	4×10^{7} cfu G45 bacteria	10 µg/ml Chloramphenicol	
180 µl saliva	20 µl plasminogen depleted plasma	4×10^{7} cfu G45 bacteria	6×10^{-7} mol $CaCl_2$	
180 µl saliva	20 µl plasminogen depleted plasma	4×10^{7} cfu G45 bacteria	6×10^{-7} mol $CaCl_2$	4 µg plasminogen
**Coagulation experiments without bacteria**				
180 µl saliva		20 µl plasma		
180 µl saliva		20 µl plasma	4 µg anti-FVII antibody	
180 µl saliva		20 µl plasma	4 µg control antibody	
180 µl saliva		20 µl plasma	10 µg H-D-Pro-Phe-Arg-CMK	
180 µl saliva		20 µl plasma	1 µg H-D-Pro-Phe-Arg-CMK	
200 µl saliva				
180 µl P<3		20 µl plasma		
180 µl S<3		20 µl plasma		
180 µl S>3 in 8.5 g/l NaCl with 1.7 mM $CaCl_2$		20 µl plasma		

180 µl S>3 in 8.5 g/l NaCl with 1.7 mM $CaCl_2$	20 µl plasma	4 µg anti-FVII antibody
180 µl S>3 in 8.5 g/l NaCl with 1.7 mM $CaCl_2$	20 µl plasma	4 µg control antibody
180 µl S>3 in 8.5 g/l NaCl with 1.7 mM $CaCl_2$	20 µl plasma	10 µg H-D-Pro-Phe-Arg-CMK
180 µl S<3	20 µl plasma	4 µg anti-FVII antibody
180 µl S<3	20 µl plasma	10 µg H-D-Pro-Phe-Arg-CMK
47 µl P<3	20 µl plasma	133 µl 1.7 mM $CaCl_2$
180 µl S<3	20 µl plasma	1.3 mg NaCl
180 µl 8.5 g/l NaCl with 1.7 mM $CaCl_2$	20 µl plasma	
180 µl 2.24 g/l NaCl with 1.7 mM $CaCl_2$	20 µl plasma	
180 µl S>3 in 2.24 g/l NaCl with 1.7 mM $CaCl_2$	20 µl plasma	

Supplementary table S2

Uniprot IDs		Protein name	Protein group*	Replicate intensity values**						Median values per replicate group		Fold_change	Multiple t-tests***	
				Pellet+bacteria_1	Pellet+bacteria_2	Pellet+bacteria_3	Pellet-bacteria_1	Pellet-bacteria_2	Pellet-bacteria_3	Pellet+bacteria	Pellet-bacteria		Discovery	P value
P02679	FIBG_HUMAN	Fibrinogen gamma chain	Fibrinogen	24890275442	23676030464	21322458676	20660043526	20750876731	19326177968	23676030464	20660043526	1.1	*	3.13753E-22
P02671	FIBA_HUMAN	Fibrinogen alpha chain	Ignored	16448471456	19207680632	17753730792	16895346332	19946099494	20868556508	17753730792	19946099494	0.9	*	2.86753E-06
P02675	FIBB_HUMAN	Fibrinogen beta chain	Ignored	20823032608	21567692352	17042739392	16439680412	19365392232	19394666144	20823032608	19365392232	1.1	*	4.04705E-06
P12273	PIP_HUMAN	Prolactin-inducible protein	Prolactin-inducible protein	3284497492	3654360064	6468563040	1257623898	1693752172	2555933602	3654360064	1693752172	2.2	*	3.1272E-17
P04003	C4BPA_HUMAN	C4b-binding protein alpha chain	Complement	634949876	807512000	495076992	752882276	691620808	333200132	634949876	691620808	0.9		0.86079
P02765	FETUA_HUMAN	Fetuin-A (Alpha-2-HS-glycoprotein)	Fetuin-A	76799200	931823000	2356179344	587414016	2246378232	2284734588	931823000	2246378232	0.4		0.0546614
Q96T23	RSF1_HUMAN	Remodeling and spacing factor 1	Other	425894016	395143008	402835008	360361984	495096000	607737984	402835008	495096000	0.8		0.792876
P02751	FINC_HUMAN	Fibronectin	Other	407876000	198554000	210608000	339278580	215256000	154663300	210608000	215256000	1.0		0.905814
P02808	STAT_HUMAN	Statherin	Statherin	319751008	3375089920	2194439936	324864992	3391170048	1316800000	2194439936	1316800000	1.7		0.347536
P02656	APOC3_HUMAN	Apolipoprotein C-III	Apolipoprotein	408012000	83549504	92394096	291076000	79444500	260812000	92394096	260812000	0.4		0.958543
Q9UGM3	DMBT1_HUMAN	Deleted in malignant brain tumors 1 protein	Other	478385608	261562200	206287008	255478800	90731504	105355000	261562200	105355000	2.5		0.587345
P02768	ALBU_HUMAN	Serum albumin	Albumin	14091902764	15581688896	13638298804	227582000	529157300	207256000	14091902764	227582000	61.9	*	0
P02743	SAMP_HUMAN	Serum amyloid P-component	Other	232312992	327372000	247570000	209192000	251264000	171228000	247570000	209192000	1.2		0.847247
P00734	THRB_HUMAN	Prothrombin	Coagulation	238268992	200765700	410772992	169902000	156830700	338744700	238268992	169902000	1.4		0.839727
P04004	VTNC_HUMAN	Vitronectin	Other	241568992	503063584	815108416	155686000	297561296	389132992	503063584	297561296	1.7		0.431339
P02647	APOA1_HUMAN	Apolipoprotein A-I	Apolipoprotein	221818000	237618000	227336000	137024000	149013200	80992000	227336000	137024000	1.7		0.725736
P01024	CO3_HUMAN	Complement C3	Complement	283546508	608782976	736029504	136968992	234752000	329588800	608782976	234752000	2.6		0.309266
P01036	CYTS_HUMAN	Cystatin-S	Other	61665900	129033000	363960700	136628992	231996020	690288992	129033000	231996020	0.6		0.580126
P61626	LYSC_HUMAN	Lysozyme C	Other	168874000	147166000	913154086	123845000	183714000	1056056988	168874000	183714000	0.9		0.882749
P02747	C1QC_HUMAN	Complement C1q subcomponent subunit C	Complement	151069804	318014016	187180000	122563100	203326000	43776900	187180000	122563100	1.5		0.753186
P02745	C1QA_HUMAN	Complement C1q subcomponent subunit A	Complement	167714400	229972000	118122000	117702500	175649496	34550500	167714400	117702500	1.4		0.836662
P0CG04	LAC1_HUMAN	Ig lambda-1 chain C regions	Other	2685289912	2486481936	2379062880	106629000	120181000	86218400	2486481936	106629000	23.3	*	8.11688E-15
P0C0L5	CO4B_HUMAN	Complement C4-B	Complement	180776992	221800992	136395008	101981000	129516000	56056700	180776992	101981000	1.8		0.782665
P02746	C1QB_HUMAN	Complement C1q subcomponent subunit B	Complement	193348400	295548800	156252508	97997934	119808380	42025900	193348400	97997934	2.0		0.672486
P07225	PROS_HUMAN	Vitamin K-dependent protein S	Other	83270304	87554400	72445904	97688304	87801900	53428000	83270304	87801900	0.9		0.99619
P01876	IGHA1_HUMAN	Ig alpha-1 chain C region	Other	319955008	409568000	412205992	95380096	62531600	125063000	409568000	95380096	4.3		0.346239
P15515	HIS1_HUMAN	Histatin-1	Other	82596400	183530000	1098630016	93948304	310062016	419511361	183530000	310062016	0.6		0.552672
P06702	S10A9_HUMAN	Protein S100-A9 (Calgranulin-B)	Other	123848600	58977200	77187898	74218100	31388500	67831200	77187898	67831200	1.1		0.924323
P05109	S10A8_HUMAN	Protein S100-A8 (Calgranulin-A)	Other	70972304	51963900	93592300	71430600	14504130	50370300	70972304	50370300	1.4		0.929861
P01857	IGHG1_HUMAN	Ig gamma-1 chain C region	IgG heavy chain	4737282292	11640812892	9805694920	69911200	90220900	106469000	9805694920	90220900	108.7	*	0
P20851	C4BPB_HUMAN	C4b-binding protein beta chain	Ignored	65789800	114451000	48834900	64796670	96842600	34605170	65789800	64796670	1.0		0.971265
P0C0L4	CO4A_HUMAN	Complement C4-A	Complement	87095200	98058096	53139500	52490300	86703600	31095900	87095200	52490300	1.7		0.940523
P02652	APOA2_HUMAN	Apolipoprotein A-II	Apolipoprotein	36468710	85421626	83517336	52257330	43274780	38751300	83517336	43274780	1.9		0.937799
P00488	F13A_HUMAN	Coagulation factor XIII A chain	Coagulation	56593700	53519500	56667300	46811100	32586720	35105800	56593700	35105800	1.6		0.95426
P00736	C1R_HUMAN	Complement C1r subcomponent	Complement	67227800	45512500	53779200	45168000	51798600	12558800	53779200	45168000	1.2		0.950138
P02649	APOE_HUMAN	Apolipoprotein E	Apolipoprotein	65260500	108297000	90137104	42239600	40036200	53821800	90137104	42239600	2.1		0.888662
P04114	APOB_HUMAN	Apolipoprotein B-100	Apolipoprotein	130128000	98247300	123717000	42066400	24124400	38382300	123717000	38382300	3.2		0.785954
P01591	IGJ_HUMAN	Immunoglobulin J chain	Other	29684600	43887600	24096500	37315860	45003800	31315980	29684600	37315860	0.8		0.986023
Q9UK55	ZPI_HUMAN	Protein Z-dependent protease inhibitor	Other	9472430	16922860	24822900	37253500	5080730	15850300	16922860	15850300	1.1		0.993901
P09871	C1S_HUMAN	Complement C1s subcomponent	Complement	47434200	42847600	31221300	33574400	34895900	7931600	42847600	33574400	1.3		0.960533
Q12986	NFX1_HUMAN	Transcriptional repressor NF-X1	Other	0	0	0	33132900	20098800	13074100	0	20098800	0.0		0.942005
P10909	CLUS_HUMAN	Clusterin	Other	18275000	53326400	42178200	29842200	41223200	23978600	42178200	29842200	1.4		0.983599
P01009	A1AT_HUMAN	Alpha-1-antitrypsin	Other	61728000	60763700	48694100	29167200	35380800	22167000	60763700	29167200	2.1		0.926158
P01023	A2MG_HUMAN	Alpha-2-macroglobulin	Other	867530776	784829316	817903084	28114200	29537800	25323800	817903084	28114200	29.1		0.00898211
P07358	CO8B_HUMAN	Complement component C8 beta chain	Complement	50150200	79474400	98406000	27590300	26911610	6544790	79474400	26911610	3.0		0.854633
P02655	APOC2_HUMAN	Apolipoprotein C-II	Apolipoprotein	23794300	30817650	25036100	27289800	17842400	26969300	25036100	26969300	0.9		0.993394
Q8WUF5	IASPP_HUMAN	RelA-associated inhibitor	Other	7368420	7384320	8238030	25512900	9062430	18527100	7384320	18527100	0.4		0.973644
P04264	K2C1_HUMAN	Keratin, type II cytoskeletal 1	Other	37048400	166276992	80740800	24803500	31236400	28383900	80740800	28383900	2.8		0.826617
P02748	CO9_HUMAN	Complement component C9	Complement	128946000	210324992	176659008	24188400	41468000	35752600	176659008	35752600	4.9		0.649272
P59665	DEF1_HUMAN	Neutrophil defensin 1	Other	96753896	11329000	141746726	22044460	22259800	56135350	96753896	22259800	4.3		0.869804
P04406	G3P_HUMAN	Glyceraldehyde-3-phosphate dehydrogenase	Other	67805650	91550200	48555800	21814400	12895500	35004700	67805650	21814400	3.1		0.879481
P08519	APOA_HUMAN	Apolipoprotein (la)	Apolipoprotein	27864100	15831500	22045600	21301300	7441510	15313700	22045600	15313700	1.4		0.981018
P28325	CYTD_HUMAN	Cystatin-D	Other	11576850	22193500	0	21285490	15689900	10380200	11576850	15689900	0.7		0.988108
P01859	IGHG2_HUMAN	Ig gamma-2 chain C region	IgG heavy chain	1220475888	1323965584	1141604312	20306400	43709600	19615100	1220475888	20306400	60.1	*	8.4911E-05
O43866	CD5L_HUMAN	CD5 antigen-like	Other	21888400	24347200	7869735	19861100	29766400	10749400	21888400	19861100	1.1		0.99451
P01037	CYTN_HUMAN	Cystatin-SN	Other	48644050	58568700	79832496	19791600	18829900	66965700	58568700	19791600	3.0		0.928784
Q96DR5	BPIA2_HUMAN	BPI fold-containing family A member 2	Other	23534900	43167400	47869900	19352600	18080200	42299400	43167400	19352600	2.2		0.969507
P00742	FA10_HUMAN	Coagulation factor X	Coagulation	0	25063700	119488000	17754000	29644900	82185696	25063700	29644900	0.8		0.986898
P08603	CFAH_HUMAN	Complement factor H	Complement	25829000	45103600	50489700	17378700	26277800	29744800	45103600	26277800	1.7		0.95798
P02810	PRPC_HUMAN	Salivary acidic proline-rich phosphoprotein 1/2	Other	0	1848994852	1194923016	16997300	2300392882	1614718952	1194923016	1614718952	0.7		0.32997
P13645	K1C10_HUMAN	Keratin, type I cytoskeletal 10	Other	26704900	143767820	68225900	15936567	22236310	25381070	68225900	22236310	3.1		0.847613
P35527	K1C9_HUMAN	Keratin, type I cytoskeletal 9	Other	4449090	56699900	62735700	15683200	17006500	14612300	56699900	15683200	3.6		0.933036
P02654	APOC1_HUMAN	Apolipoprotein C-I	Apolipoprotein	22144650	20701000	31713000	15665200	13711300	13026855	22144650	13711300	1.6		0.971856
Q16610	ECM1_HUMAN	Extracellular matrix protein 1	Other	27993800	20349800	5399305	15227100	11859200	7264160	20349800	11859200	1.7		0.983025
P07357	CO8A_HUMAN	Complement component C8 alpha chain	Ignored	53525400	86108600	72801100	14810900	16755500	14556700	72801100	14810900	4.9		0.855211
P27918	PROP_HUMAN	Properdin	Complement	56207800	80003600	71918600	13674900	27105000	14986800	71918600	14986800	4.8		0.867236
P05155	IC1_HUMAN	Plasma protease C1 inhibitor	Other	78222500	27884600	28116700	13671900	30152700	5250990	28116700	13671900	2.1		0.925567
P80511	S10AC_HUMAN	Protein S100-A12	Other	0	0	0	12939100	0	0	0	0			0.988673
P63261	ACTG_HUMAN	Actin, cytoplasmic 2	Other	23590100	0	18454500	12271900	0	12916530	18454500	12271900	1.5		0.985244
P02760	AMBP_HUMAN	Protein AMBP	Other	9254740	17815300	12210900	11946100	8996060	20735800	12210900	11946100	1.0		0.997902
Q99973	TEP1_HUMAN	Telomerase protein component 1	Other	0	0	12557500	11767000	10716200	12109100	0	11767000	0.0		0.980712
P04275	VWF_HUMAN	von Willebrand factor	Coagulation	12160000	21677550	4271830	11495500	4262790	5709300	12160000	5709300	2.1		0.985432
P03973	SLPI_HUMAN	Antileukoproteinase	Other	47908500	15027400	165935008	11244800	2590990	67815000	47908500	11244800	4.3		0.871678
P04196	HRG_HUMAN	Histidine-rich glycoprotein	Other	96915400	22216900	79905296	11212420	9775900	15224500	79905296	11212420	7.1		0.858214
P01042	KNG1_HUMAN	Kininogen-1	Other	27861600	20443200	40155600	10918500	16094200	33635300	27861600	16094200	1.7		0.975656
P12259	FA5_HUMAN	Coagulation factor V	Coagulation	0	16960875	18609700	10481990	10463495	7108850	16960875	10463495	1.6		0.99342
P02741	CRP_HUMAN	C-reactive protein	Other	9087180	36009750	13393050	10410300	10102000	7126360	13393050	10102000	1.3		0.972997
P10643	CO7_HUMAN	Complement component C7	Complement	71551296	108174000	76278896	10355000	17329700	10903000	76278896	10903000	7.0		0.81146
P13671	CO6_HUMAN	Complement component C6	Complement	64440400	76726304	70709696	9859620	9912480	25704850	70709696	9912480	7.1		0.855136
P07360	CO8G_HUMAN	Complement component C8 gamma chain	Ignored	32724500	100023100	80095400	9687380	25723200	9431140	80095400	9687380	8.3		0.853757
P01031	CO5_HUMAN	Complement C5	Complement	66747900	108052000	109293636	8300930	13966000	10539300	108052000	10539300	10.3		0.782777
P08697	A2AP_HUMAN	Alpha-2-antiplasmin	Other	10584865	28913750	0	8242600	5387225	4762785	10584865	5387225	2.0		0.981525
Q08188	TGM3_HUMAN	Protein-glutamine gamma-glutamyltransferase E)	Other	9132600	16994900	27709900	8191190	3240760	5763000	16994900	5763000	2.9		0.967931
P19652	A1AG2_HUMAN	Alpha-1-acid glycoprotein 2	Other	11170740	10356830	13496925	7845935	6524000	5676545	11170740	6524000	1.7		0.986888
P23280	CAH6_HUMAN	Carbonic anhydrase 6	Other	9659450	13139705	25726000	7659600	7867660	22541800	13139705	7867660	1.7		0.990847
P35908	K22E_HUMAN	Keratin, type II cytoskeletal 2 epidermal	Other	44166000	86872096	66324800	7544390	57097700	7953970	66324800	7953970	8.3		0.891116
P06727	APOA4_HUMAN	Apolipoprotein A-IV	Apolipoprotein	16498400	13866000	62299950	7350060	7926660	4493490	16498400	7350060	2.2		0.936254
P01833	PIGR_HUMAN	Polymeric immunoglobulin receptor	Other	42465400	29277900	51305000	7260370	22027900	24471200	42465400	22027900	1.9		0.939401
P08493	MGP_HUMAN	Matrix Gla protein	Other	0	8755740	21302500	6883370	21703200	23481200	8755740	21703200	0.4		0.980734
Q14515	SPRL1_HUMAN	SPARC-like protein 1	Other	25057800	20731100	17310400	6593220	7053330	20594900	20731100	7053330	2.9		0.974741
P02787	TRFE_HUMAN	Serotransferrin	Other	50120530	32071500	33962500	6463115	16698200	5229790	33962500	6463115	5.3		0.923288
Q03591	FHR1_HUMAN	Complement factor H-related protein 1	Complement	30202800	29532600	39891400	6291660	7146770	13678700	30202800	7146770	4.2		0.93659
P80303	NUCB2_HUMAN	Nucleobindin-2	Other	14032020	9230260	28934200	6285100	2002940	12806400	14032020	6285100	2.2		0.972778
P22891	PROZ_HUMAN	Vitamin K-dependent protein Z	Other	97044846	5173455	44053800	5640730	13564000	19633100	44053800	13564000	3.2		0.906166
P01019	ANGT_HUMAN	Angiotensinogen	Other	7453990	4611900	10790200	5389285	4725430	0	7453990	4725430	1.6		0.988846
P00738	HPT_HUMAN	Haptoglobin	Other	70774704	51832900	64737300	5236175	21874900	10303500	64737300	10303500	6.3		0.869337
P01008	ANT3_HUMAN	Antithrombin-III	Coagulation	84259296	113315654	107437000	4849465	3978250	15696600	107437000	4849465	22.2		0.758282
P27169	PON1_HUMAN	Serum paraoxonase/arylesterase 1	Other	0	0	0	4671080	620919	3647490	0	3647490	0.0		0.992174
P22792	CPN2_HUMAN	Carboxypeptidase N subunit 2	Other	15892700	14475700	17643250	4654730	5860630	4786985	15892700	4786985	3.3		0.971371
P19823	ITIH2_HUMAN	Inter-alpha-trypsin inhibitor heavy chain H2	Other	3434260	0	0	3971160	6582530	2998650	0	3971160	0.0		0.991143
P23142	FBLN1_HUMAN	Fibulin-1	Other	14906900	18762600	0	3897410	5438020	1818330	14906900	3897410	3.8		0.980291
P02766	TTHY_HUMAN	Transthyretin	Other	0	13170100	6534440	3879080	824785	0	6534440	824785	7.9		0.986869
P61769	B2MG_HUMAN	Beta-2-microglobulin	Other	0	0	0	3713270	6776800	4628440	0	4628440	0.0		0.986765
P18428	LBP_HUMAN	Lipopolysaccharide-binding protein	Other	2315480	0	0	3701980	2202430	0	0	2202430	0.0		0.996858
Q96I82	KAZD1_HUMAN	Kazal-type serine protease inhibitor domain-containing protein	Other	0	0	0	3585930	0	0	0	0			0.996861
P04070	PROC_HUMAN	Vitamin K-dependent protein C	Other	31540800	29657700	48959400	3422780	8878910	29877700	31540800	8878910	3.6		0.940545
P35542	SAA4_HUMAN	Serum amyloid A-4 protein	Other	4741250	0	0	3247530	0	3361760	0	3247530	0.0		0.998365
P14618	KPYM_HUMAN	Pyruvate kinase PKM	Other	5941150	16102610	4383320	2967315	4253045	3089670	5941150	3089670	1.9		0.985891
P02788	TRFL_HUMAN	Lactotransferrin	Other	67293000	28035900	67327400	2840830	1466320	9205360	67293000	2840830	23.7		0.870016
O95445	APOM_HUMAN	Apolipoprotein M	Apolipoprotein	0	0	0	2812350	0	4016510	0	2812350	0.0		0.994022
P04745	AMY1_HUMAN	Alpha-amylase 1	Other	6917850	45293900	59551800	2713960	11396800	12173950	45293900	11396800	4.0		0.925279
P06396	GELS_HUMAN	Gelsolin	Other	0	0	0	2699540	3915430	3601010	0	3601010	0.0		0.991057
Q9HC84	MUC5B_HUMAN	Mucin-5B	Other	59489535	13828600	90315904	2699145	10589700	86187800	59489535	10589700	5.6		0.943881
P02790	HEMO_HUMAN	Hemopexin	Other	11358070	0	11992000	2419410	4494890	0	11358070	2419410	4.7		0.985612
P15169	CBPN_HUMAN	Carboxypeptidase N catalytic chain	Other	0	0	0	2250695	2990640	2095400	0	2250695	0.0		0.993577
P00740	FA9_HUMAN	Coagulation factor IX	Coagulation	73700000	76992600	25424900	2108120	4071360	16714900	73700000	4071360	18.1		0.866494
P15153	RAC2_HUMAN	Ras-related C3 botulinum toxin substrate 2	Other	0	0	0	2067660	0	0	0	0			0.99819
P02763	A1AG1_HUMAN	Alpha-1-acid glycoprotein 1	Other	64325200	121608000	101445000	1998590	5639290	0	101445000	1998590	50.8		0.758906
P08779	K1C16_HUMAN	Keratin, type I cytoskeletal 16	Other	0	0	0	1927750	3002700	0	0	1927750	0.0		0.995684
P02774	VTDB_HUMAN	Vitamin D-binding protein	Other	19911570	0	8303990	1877635	3404250	1051898	8303990	1877635	4.4		0.980846
P23284	PPIB_HUMAN	Peptidyl-prolyl cis-trans isomerase B	Other	20763900	22746250	54353800	1798950	6600080	35165100	22746250	6600080	3.4		0.952492
Q08380	LG3BP_HUMAN	Galectin-3-binding protein	Other	13772160	9324240	0	1791980	3467530	2937050	9324240	2937050	3.2		0.986957
O14791	APOL1_HUMAN	Apolipoprotein L1	Other	15822700	13652400	0	1765995	3647790	0	13652400	1765995	7.7		0.978938
Q14533	KRT81_HUMAN	Keratin, type II cuticular Hb1	Other	0	0	8758400	1717870	2058930	1994350	0	1994350	0.0		0.997385
P22079	PERL_HUMAN	Lactoperoxidase	Other	13002900	0	11558305	1637220	4211020	6283220	11558305	4211020	2.7		0.989119
P07237	PDIA1_HUMAN	Protein disulfide-isomerase	Other	0	0	6288720	1342330	1957610	4826820	0	1957610	0.0		0.998391
P00751	CFAB_HUMAN	Complement factor B	Complement	16883200	25012100	27308300	1221790	1812935	1712275	25012100	1712275	14.6		0.94362
P54108	CRIS3_HUMAN	Cysteine-rich secretory protein 3	Other	0	7013280	170524000	983179	6745710	34041800	7013280	6745710	1.0		0.881585
Q86YZ3	HORN_HUMAN	Hornerin	Other	0	1451860	2033800	934333.5	1496593	1633600	1451860	1496593	1.0		0.999493
P04217	A1BG_HUMAN	Alpha-1B-glycoprotein	Other	0	0	0	751535	0	0	0	0			0.999342
P03950	ANGI_HUMAN	Angiogenin	Other	0	0	0	745383	1604820	4496130	0	1604820	0.0		0.994007
P27797	CALR_HUMAN	Calreticulin	Other	6148310	0	0	726444	2866600	1936570	0	1936570	0.0		0.999458
Q9UBG3	CRNN_HUMAN	Cornulin	Other	0	0	0	724548	3415645	0	0	724548	0.0		0.996376
P07900	HS90A_HUMAN	Heat shock protein HSP 90-alpha	Other	42986500	34480200	29143100	663867	0	3283150	34480200	663867	51.9		0.910316
P31947	1433S_HUMAN	14-3-3 protein sigma	Other	0	867275	2117460	69946.20313	1077510	581634	867275	581634	1.5		0.998901
P38646	GRP75_HUMAN	Stress-70 protein, mitochondrial	Other	482042816	415172692	357869698	0	0	0	415172692	0			0.168788
P06576	ATPB_HUMAN	ATP synthase subunit beta, mitochondrial	Other	239251008	363083270	295288130	0	0	0	295288130	0			0.324864
P01861	IGHG4_HUMAN	Ig gamma-4 chain C region	IgG heavy chain	194959300	265952000	214628000	0	0	0	214628000	0			0.458666
P25705	ATPA_HUMAN	ATP synthase subunit alpha, mitochondrial	Other	199033008	234741500	202174208	0	0	0	202174208	0			0.485407
Q9NYV6	RRN3_HUMAN	RNA polymerase I-specific transcription initiation factor RRN3	Other	57143200	65028500	72107000	0	0	0	65028500	0			0.831204
O00186	STXB3_HUMAN	Syntaxin-binding protein 3	Other	72643800	63456800	55996700	0	0	0	63456800	0			0.833071
P35858	ALS_HUMAN	Insulin-like growth factor-binding protein complex acid labile su	Other	23331805	62246100	50762100	0	1343170	2081340	50762100	1343170	37.8		0.884054
P06312	KV401_HUMAN	Ig kappa chain V-IV region	Other	32058600	30778500	27320250	0	0	0	30778500	0			0.921202
P00338	LDHA_HUMAN	L-lactate dehydrogenase A chain	Other	35370400	22767800	29715700	0	0	0	29715700	0			0.923209
P07195	LDHB_HUMAN	L-lactate dehydrogenase B chain	Ignored	26489300	18188800	12128400	0	0	0	18188800	0			0.950302
P05546	HEP2_HUMAN	Heparin cofactor 2	Other	0	16320000	3819240	0	4080440	0	3819240	0			0.985942
P11021	GRP78_HUMAN	78 kDa glucose-regulated protein -binding protein grp78)	Other	9052050	14552300	55114198	0	0	5012540	14552300	0			0.935546
Q16739	CEGT_HUMAN	Ceramide glucosyltransferase	Other	0	10358700	0	0	0	10626800	0	0			0.999765
P00739	HPTR_HUMAN	Haptoglobin-related protein	Other	0	9222825	0	0	8227220	7026720	0	7026720	0.0		0.99472
P25391	LAMA1_HUMAN	Laminin subunit alpha-1	Other	6055580	4545400	9978470	0	0	0	6055580	0			0.981986
P34096	RNAS4_HUMAN	Ribonuclease 4	Other	0	912254	0	0	0	2131430	0	0			0.998933
P05090	APOD_HUMAN	Apolipoprotein D	Apolipoprotein	51012000	0	95354304	0	0	0	51012000	0			0.872416
Q96AN5	TM143_HUMAN	Transmembrane protein 143	Other	37086600	0	34073000	0	0	0	34073000	0			0.937768
P01011	AACT_HUMAN	Alpha-1-antichymotrypsin	Other	24524350	0	7514280	0	0	2015960	7514280	0			0.973722
Q9BYR8	KRA31_HUMAN	Keratin-associated protein 3-1	Other	0	0	18536300	0	0	0	0	0			0.983774
P00450	CERU_HUMAN	Ceruloplasmin	Other	8975285	0	4645030	0	2170180	2179810	4645030	2170180	2.1		0.991885
P78386	KRT85_HUMAN	Keratin, type II cuticular Hb5	Other	0	0	10692225	0	0	0	0	0			0.99064
Q9BYQ2	KRA94_HUMAN	Keratin-associated protein 9-4	Other	0	0	10330300	0	0	0	0	0			0.990957
Q8N4F0	BPIB2_HUMAN	BPI fold-containing family B member 2	Other	0	0	7922690	0	0	733907	0	0			0.993707
P55268	LAMB2_HUMAN	Laminin subunit beta-2	Other	4914470	0	2412230	0	0	0	2412230	0			0.993586
P07602	SAP_HUMAN	Prosaposin	Other	0	0	3279530	0	0	2350435	0	0			0.999187
P28799	GRN_HUMAN	Granulins	Other	5300440	0	0	0	0	0	0	0			0.99536
P09429	HMGB1_HUMAN	High mobility group protein B1	Other	0	0	0	0	0	4228090	0	0			0.996299
P53634	CATC_HUMAN	Dipeptidyl peptidase 1	Other	0	0	0	0	0	3146990	0	0			0.997245
Q15323	K1H1_HUMAN	Keratin, type I cuticular Ha1	Other	0	0	2599010	0	0	0	0	0			0.997725
P07686	HEXB_HUMAN	Beta-hexosaminidase subunit beta	Other	0	0	0	0	0	2431945	0	0			0.997871
P15311	EZRI_HUMAN	Ezrin	Other	0	0	0	0	0	1571120	0	0			0.998625
Q96PD5	PGRP2_HUMAN	N-acetylmuramoyl-L-alanine amidase	Other	0	0	0	0	890544	0	0	0			0.99922
Q92743	HTRA1_HUMAN	Serine protease HTRA1	Other	0	0	0	0	0	732539	0	0			0.999359
P35556	FBN2_HUMAN	Fibrillin-2	Other	0	0	455472	0	0	0	0	0			0.999601

* Protein groups used in Fig. 1C. The ignored group contains protein chains of which intensity values were omitted in Fig. 1C.

** Proteins were identified with the Trans Proteomic Pipeline using 1% False Discovery Rate (FDR) and Label-free quantification values determined by OpenMS

*** Multiple t-test (one by row) of replicate intensity values calculated by software Graphpad Prism 6.0g assuming that all rows were sampled from populations with equal same scatter and the Q-value (FDR) set to 1%.
